# Hair tourniquet syndrome leading to nipple autoamputation in an adult female: a case report

**DOI:** 10.3389/fsurg.2025.1531270

**Published:** 2025-03-07

**Authors:** Alaa A. Alshurafa, Khaled Alshawwa

**Affiliations:** ^1^ Research Department, Palestine Red Crescent Society, Gaza, Palestine; ^2^ Healthcare Workers Watch, Gaza, Palestine; ^3^ General Surgery Department, Faculty of Medicine, Al-Azhar University, Gaza, Palestine

**Keywords:** hair tourniquet, nipple, autoamputation, case report, adult

## Abstract

This is a unique case of hair thread tourniquet syndrome (HTTS) affecting the nipple of an adult female, leading to spontaneous autoamputation, a phenomenon rarely documented in the literature for this anatomical site. A 52-year-old woman presented with changes in the color and shape of her left nipple over two days, with a piece of hair wrapped around it. Despite hair removal, her symptoms worsened, ultimately resulting in necrosis and autoamputation of the nipple. Unlike most reported cases, which involve early intervention preventing severe ischemic damage, this case highlights the rare progression of HTTS to complete tissue loss. The necrotic portion was easily excised under local anesthesia. Awareness of this condition and prompt intervention are crucial to prevent severe complications in adult HTTS.

## Introduction

Hair tourniquet syndrome (HTTS) is a surgical emergency in which hair or textile thread strangulates body appendages, risking prolonged ischemia and tissue necrosis. If untreated, it may lead to autoamputation of the affected appendage. HTTS predominantly affects pediatric patients, typically involving the fingers, toes, or genitalia, but cases in adults are rare, risk factors include cognitive or psychiatric conditions (1, 2), circumcision in males (3), and telogen effluvium (4). This report presents an unusual case of HTTS involving the nipple in an adult female, leading to autoamputation; to our knowledge, such a case has not been previously reported. Written informed consent was obtained from the patient for publication of this case report and any accompanying images. This case report was prepared in accordance with the surgical case report (SCARE) guidelines to ensure the structured and transparent reporting of surgical cases (5).

## Case presentation

A 52-year-old premenopausal female presented to our primary healthcare clinic with complaints of left nipple color and shape changes lasting for two days. The patient exhibited swelling, redness, and congestion of the left nipple. She reported seeing a piece of hair wrapped around the nipple, forming a constricting waist, which she claimed removed. Despite this, the symptoms persisted and escalated.

Key clinical concerns included significant swelling and congestion in the absence of pain, fever, discharge or other systemic symptoms. The patient had no personal or family history of breast disorders or any notable psychosocial disorders related to this presentation. She recalled previous episodes where hair wrapped around her nipple caused mild congestion and discoloration, which resolved immediately upon hair removal. However, despite hair removal, symptoms persist and escalate.

### Examination and timeline of events

**Figure 1:**
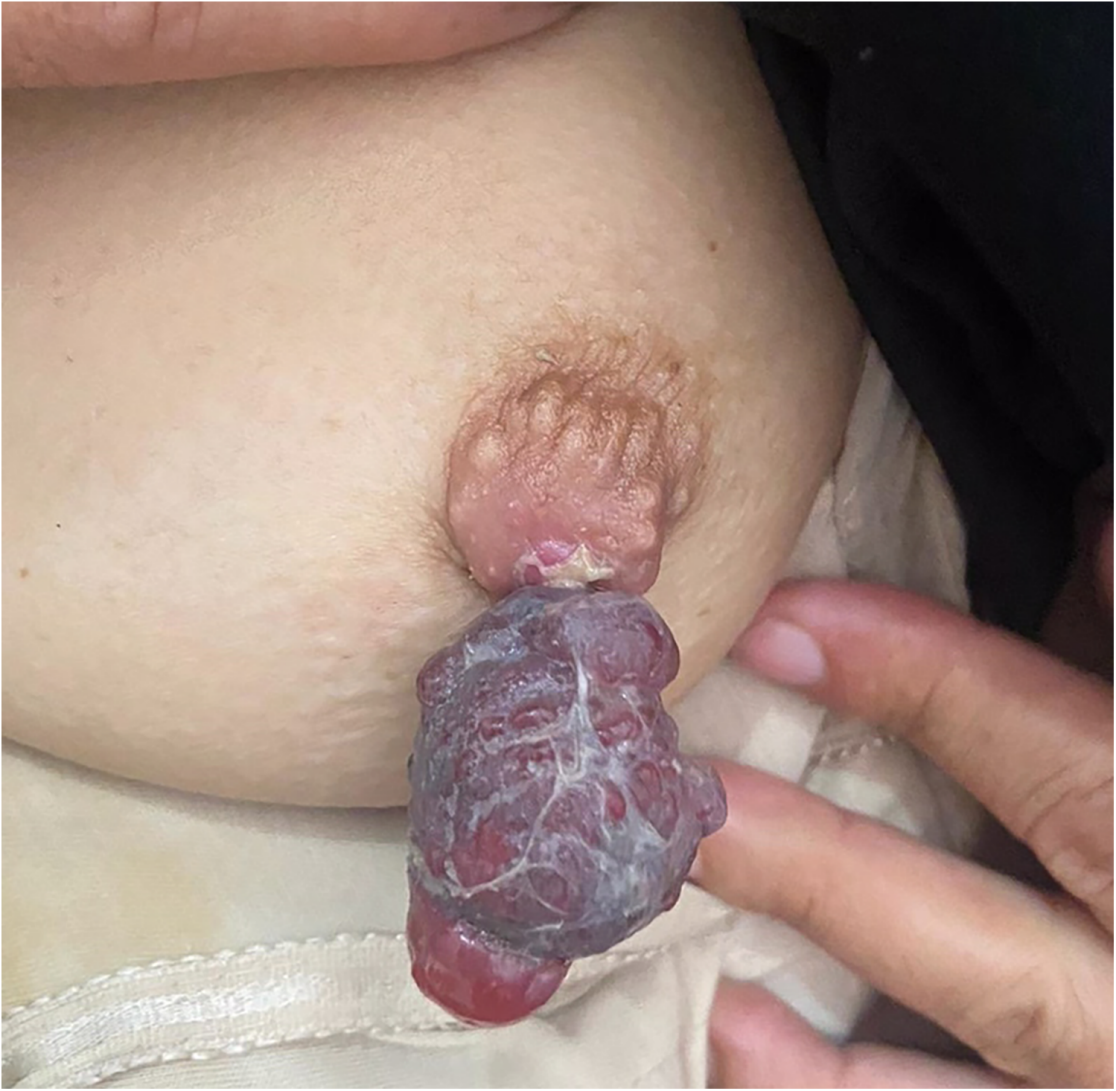
Initial presentation showing a congested and swollen left nipple.

**Figure 2:**
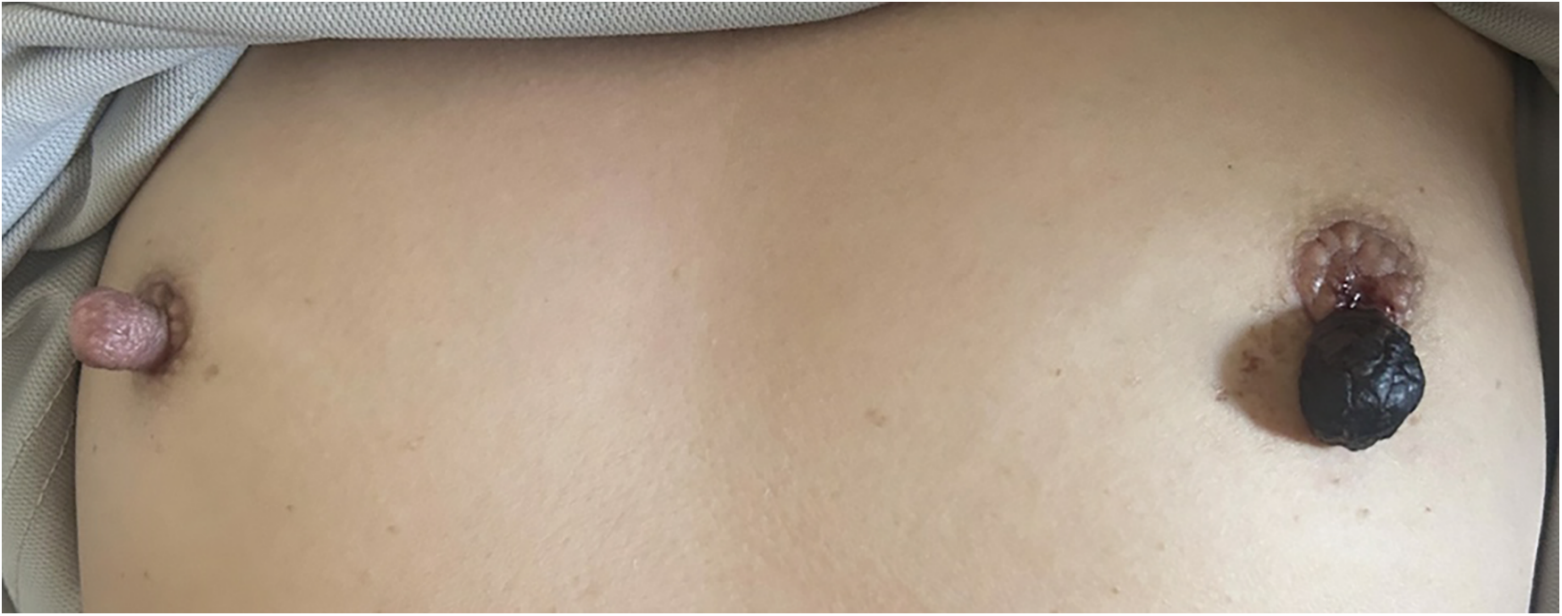
Left nipple upon representation exhibiting necrotic changes connected by a thin stalk. Note the unusual prominence of the right nipple.

**Figure 3:**
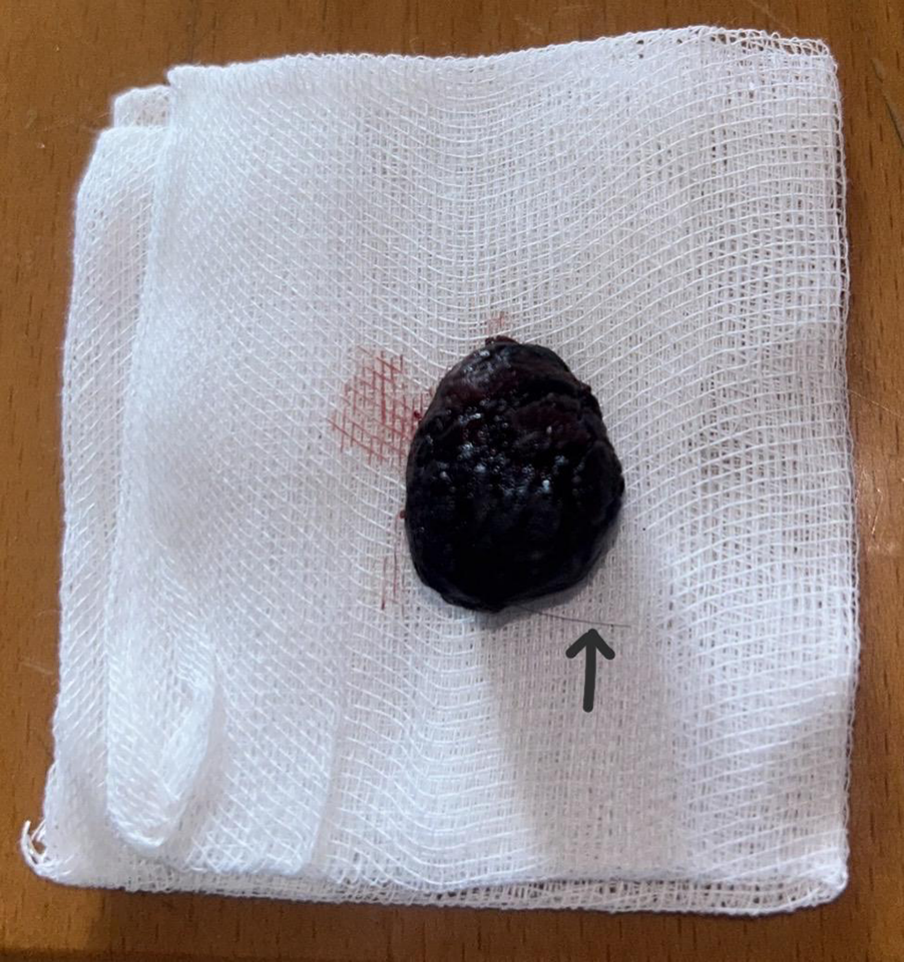
Necrotic nipple after being completely excised. Note the presence of a hair thread (black arrow).

On **Day 1**, the patient noticed initial nipple congestion and attempted to remove her constricting hair; despite her efforts, her symptoms progressed, with increased swelling and color changes. Therefore, upon physical examination, the left nipple was enlarged, with significant redness and congestion, resembling an exophytic mass(shown in Figure 1) and she was referred to the general surgery clinic. On **Day 6**, owing to safety concerns and evacuation orders related to the war on Gaza, she was unable to access the surgery clinic; by the time she presented at the general surgery clinic, the left nipple had autoamputated, with the necrotic portion connected to the breast by a small stalk, producing malodorous discharge. Careful inspection by the surgeon revealed that the hair thread constricted the necrotic nipple (shown in Figures 2, 3) the right nipple remained normal, but it was noticeable that the patient had an obviously protruded nipple (shown in Figure 2).

Both breast and axillary ultrasound were performed, which revealed completely normal findings, without any detectable masses, cysts or enlarged lymph nodes, despite the presence of visible external changes. The necrotic nipple was excised under local anesthesia using 2% lidocaine infiltration for adequate analgesia. Division of the necrotic part at the waist -made by the strangulating hair thread—was easily performed. Hemostasis was achieved with compression by a gauze for 5 min. The wound was irrigated with sterile saline then simple dressing and the specimen was planned to be sent for histopathological examination, but this was not possible because of a lack of functioning laboratories during the ongoing war in Gaza.

This case presented diagnostic challenges due to the lack of systemic symptoms such as pain or fever, as well as normal ultrasound findings despite the apparent necrosis of the nipple. The patient was diagnosed with necrosis of the left nipple, possibly secondary to ischemia induced by hair constriction.

## Post-operative follow-up

Standard postoperative management guidelines were followed, including infection prevention strategies such as antibiotic prophylaxis with a first-generation cephalosporin and advice given for routine wound care with sterile dressings. Patients with similar conditions are typically advised to avoid mechanical irritation to the surgical site and to keep the wound dry for the first 48 h. In normal circumstances, follow-up is recommended within 7–10 days to assess wound healing and detect any signs of infection or dehiscence. However, due to the ongoing war-related evacuation, follow-up was not possible in this case.

## Discussion

While HTTS is a known pediatric condition, it is rare in adults and has even fewer cases involving the nipple (6, 7). Recently, increasing numbers of HTTS cases in the adult population have been described in the literature (3, 8). The diagnosis of HTTS in adults, especially at unusual sites such as the nipple, can be complicated by normal systemic findings and minimal symptoms, as was observed here. Despite notable external changes, ultrasound imaging did not reveal underlying abnormalities. In such cases, reliance on thorough physical examination is critical when systemic or imaging indicators are inconclusive. The unique presentation in this case could be influenced by several factors. (1) Anatomical factors: The patient's pronounced nipple protrusion may have made her more susceptible to entanglement with stray hairs. (2) Hormonal influence: Premenopausal status and associated hormonal fluctuations might contribute to anatomical changes. However, previous literature has linked between hormonal changes that occurs during postpartum period and leads to telogan effluviu and perdiatric HTTS (4). (3) Environmental and social conditions: Living under challenging conditions in Gaza, where access to timely healthcare is limited, likely contributed to the delayed presentation and absence of adequate follow-up. For adult cases of HTTS, especially at nontypical sites, we recommend a high index of suspicion in cases presenting with appendage strangulation; even if symptoms are mild, thorough inspection for possible constricting agents, especially in cases with atypical locations or delayed presentation, and documentation of a protocol for similar cases could support clinicians in accurately diagnosing and managing rare presentations of HTTS, particularly in resource-limited settings.

Follow-up information was limited in this case because of ongoing challenges in accessing healthcare and disruption of healthcare system during the Gaza conflict. This limitation impacts the case's contribution to the literature, as details on healing progression or possible recurrence remain unknown. Although psychosocial factors were ruled out in this case, conditions such as trichotillomania or other behavioral components should be considered for similar presentations (2, 9). Self-attempted hair removal by the patient could indicate an underlying behavioral pattern, although this was not explored further because of the collapse of the healthcare system. The inability to conduct a histopathological examination restricted the diagnostic depth of this case. Without this, uncertainty remains regarding the full scope of contributing factors. Differential diagnoses, including exophytic tumors and trauma-related necrosis, were considered, but HTTS remains the most plausible explanation given the clinical presentation. Future cases would benefit from including histopathology to eliminate differential diagnoses and better understand the impact of constrictive injuries on specific tissue types.

Nipple-areola reconstruction (NAR) plays an essential role in restoring symmetry and aesthetics following nipple loss. These methods include nipple sharing, local flaps, augmentation grafting using autologous or heterologous materials, prosthesis or 3D tattoos, The starting point should take into consideration laterality. When nipple reconstruction is unilateral, the contralateral side should be used as template. When the unaffected nipple has a projection >1 cm, consider using nipple sharing, otherwise use local flaps (10). We believe nipple sharing would have been the best approach to our patient, because this method can ameliorate the projection of the right nipple, lowering the chance of fewer incidence of similar condition. However, these procedures are currently inaccessible in Gaza due to limited surgical resources amid the ongoing conflict. The patient was informed of future reconstruction options, contingent on improved healthcare availability.

## Conclusion

This case underscores the importance of early identification and management of HTTS, even in adult patients and at atypical sites. Given the challenges posed by diagnostic limitations and resource constraints in conflict zones, there is a need for heightened clinical vigilance and adaptability in such cases. Increased awareness among clinicians and a structured diagnostic protocol could improve outcomes for adult HTTS patients, particularly those presenting at unusual sites such as the nipple.

## Data Availability

The original contributions presented in the study are included in the article/Supplementary Material, further inquiries can be directed to the corresponding author.

## References

[1] Barton DJ Sloan GM Nichter LS Reinisch JF. Hair-thread tourniquet syndrome. Pediatrics. (1988) 82(6):925–8. 3186385

[2] Miller RR Baker WE Brandeis GH. Hair-thread tourniquet syndrome in a cognitively impaired nursing home resident. Adv Skin Wound Care. (2004) 17(7):351–2. 10.1097/00129334-200409000-00014 15343084

[3] Sallami S Ben Rhouma S Cherif K Noura Y. Hair-thread tourniquet syndrome in an adult penis: case report and review of literature. Urol J. (2013) 10(2):915–8. 23801480

[4] Strahlman RS. Toe tourniquet syndrome in association with maternal hair loss. Pediatrics. (2003) 111(3):685–7. 10.1542/peds.111.3.685 12612260

[5] Sohrabi C Mathew G Maria N Kerwan A Franchi T Agha RA The SCARE 2023 guideline: updating consensus surgical CAse REport (SCARE) guidelines. Int J Surg. (2023) 109(5):1136–40. 10.1097/JS9.0000000000000373 37013953 PMC10389401

[6] Golshevsky J Chuen J Tung PH. Hair-thread tourniquet syndrome. J Paediatr Child Health. (2005) 41(3):154–5. 10.1111/j.1440-1754.2005.00569.x 15790330

[7] Köroğlu M Özdeş HU Özbey R Yılmaz Ö Ergen E Oklu Y Hair tourniquet syndrome of toe. Foot Ankle Surg. (2023) 29(6):462–5. 10.1016/j.fas.2023.06.008 37393127

[8] Okur OM Coskun A Kayipmaz AE Ozbay S Kavalci C Kocalar UG. Hair-thread tourniquet syndrome originating from a haemangioma in an adult patient. J Pak Med Assoc. (2016) 66(7):896–7. 27427144

[9] Sudhan ST Gupta S Plutarco C. Toe-tourniquet syndrome–accidental or intentional? Eur J Pediatr. (2000) 159(11):866. 10.1007/PL00008357 11079205

[10] Paolini G Firmani G Briganti F Sorotos M Santanelli di Pompeo F. Guiding nipple-areola complex reconstruction: literature review and proposal of a new decision-making algorithm. Aesthetic Plast Surg. (2021) 45(3):933–45. 10.1007/s00266-020-02047-9 33216178 PMC8144123

